# Endogenous cAMP elevation in *Brassica napus* causes changes in phytohormone levels

**DOI:** 10.1080/15592324.2024.2310963

**Published:** 2024-02-05

**Authors:** Tianming Li, Wenjing Jia, Song Peng, Yanhui Guo, Jinrui Liu, Xue Zhang, Panyu Li, Hanfeng Zhang, Ruqiang Xu

**Affiliations:** a School of Agricultural Sciences, Zhengzhou University, Zhengzhou, Henan, China; b Zhengzhou Research Base, State Key Laboratory of Cotton Biology, Zhengzhou University, Zhengzhou, China; c Center for Excellence in Molecular Plant Sciences, Chinese Academy of Sciences, Shanghai, China

**Keywords:** Adenylate cyclase, cyclic AMP, phytohormone, signaling, *Brassica napus*

## Abstract

In higher plants, the regulatory roles of cAMP (cyclic adenosine 3′,5′-monophosphate) signaling remain elusive until now. Cellular cAMP levels are generally much lower in higher plants than in animals and transiently elevated for triggering downstream signaling events. Moreover, plant adenylate cyclase (AC) activities are found in different moonlighting multifunctional proteins, which may pose additional complications in distinguishing a specific signaling role for cAMP. Here, we have developed rapeseed (*Brassica napus* L.) transgenic plants that overexpress an inducible plant-origin AC activity for generating high AC levels much like that in animal cells, which served the genetic model disturbing native cAMP signaling as a whole in plants. We found that overexpression of the soluble AC activity had significant impacts on the contents of indole-3-acetic acid (IAA) and stress phytohormones, i.e. jasmonic acid (JA), abscisic acid (ABA), and salicylic acid (SA) in the transgenic plants. Acute induction of the AC activity caused IAA overaccumulation, and upregulation of *TAA1* and *CYP83B1* in the IAA biosynthesis pathways, but also simultaneously the hyper-induction of *PR4* and *KIN2* expression indicating activation of JA and ABA signaling pathways. We observed typical overgrowth phenotypes related to IAA excess in the transgenic plants, including significant increases in plant height, internode length, width of leaf blade, petiole length, root length, and fresh shoot biomass, as well as the precocious seed development, as compared to wild-type plants. In addition, we identified a set of 1465 cAMP-responsive genes (CRGs), which are most significantly enriched in plant hormone signal transduction pathway, and function mainly in relevance to hormonal, abiotic and biotic stress responses, as well as growth and development. Collectively, our results support that cAMP elevation impacts phytohormone homeostasis and signaling, and modulates plant growth and development. We proposed that cAMP signaling may be critical in configuring the coordinated regulation of growth and development in higher plants.

## 1. Introduction

Cyclic AMP (cyclic adenosine 3′,5′-monophosphate; 3’,5’-cAMP; cAMP) is the prototypical second messenger discovered over 60 years ago, eventually leading to a Nobel Prize. It was initially found to play fundamental regulatory roles in cellular responses to many hormones and neurotransmitters in animals.^1^ cAMP has been well demonstrated to function across different living organisms from bacteria to animals decades ago,^2–5^ but it remains poorly understood in higher plants until now. For a long period until the early 1990s, the occurrence and functions of cAMP in higher plants have been controversial due to the findings of low and even undetectable cellular cAMP levels.^6,7^ In recent years, increasing molecular studies have shown that cAMP may be implicated in many biological processes from plant growth and development to sensing and response to environmental conditions,^8–10^ although little is known about cAMP signaling cascades in plants.

A series of signaling events is initiated by the elevation of intracellular cAMP levels which are regulated by the balance between the activities of adenylyl cyclase (AC) to synthesize cAMP and cyclic nucleotide phosphodiesterase (PDE) for its degradation.^11^ However, the induction of cAMP signaling is largely dependent upon AC activation to rapidly increase cAMP.^12^ In mammals, 10 different genes each encode either the membrane-bound AC (mAC) or the soluble AC (sAC) that have the enzymatic activity as their only function^13^; in contrast, all AC genes identified so far in higher plants code for complex multifunctional proteins,^8,14^ featuring the so-called moonlighting proteins in which a single polypeptide chain exhibits more than one different function.^15,16^ For example, both AtKUP5 and AtKUP7 each contain a functional cytosolic AC catalytic center, but they are well-defined K^+^-uptake transporters in *Arabidopsis thaliana*.^17,18^ The other ACs identified in Arabidopsis include the leucine-rich repeat (LRR) protein AtLRRAC1 implicated in immune responses,^19^ AtCIAP annotated with a role in clathrin assembly and endocytosis,^20^ and the pentatricopeptide repeat-containing protein AtPPR-AC being responsible for interacting with RNA and facilitating its processing.^21^ More recently, AC activities were determined in a putative disease-resistance RPP13-like protein 3 (ZmRPP13-LK3) involving abscisic acid-mediated resistance to heat stress in *Zea mays*.^22^ All the above-described genes each not only function as an AC but also have other distinctive functions. The functional divergence of AC genes in higher plants may make it complicated to distinguish a specific signaling role for cAMP.

Exogenous application of cAMP in higher plants had been found to exert interactive effects with phytohormones in earlier studies.^23–27^ Coincidentally, we have recently noted that endogenous cAMP elevation caused alteration of phytohormone contents in *Arabidopsis*.^10^ Thus, cAMP may function in close association with phytohormones in higher plants. This notion has been supported by most recent findings that TRANSPORT INHIBITOR RESPONSE 1 (TIR1)/AUXIN-SIGNALING F-BOX (AFB) auxin receptors possess AC activity and are stimulated by auxin, together with Aux/IAAs, to produce cAMP, which is important for its function in root growth regulation and transcriptional response.^28^ Plants rely on a set of phytohormones to regulate every aspect of their biology.^29^ Recently, it became evident that phytohormone changes are closely associated with yield production and adaptation to environmental conditions in crop plants.^30–33^ In addition, a report revealed that the introduction of *Lonicera japonica*, a traditional Chinese medicinal plant, from the geo‑authentic areas to nonauthentic production areas resulted in significant changes of various phytohormones in flower buds, which altered its medicinal quality.^34^ It will be of significance to understand the part of cAMP to modulate phytohormones in crop plants for improving productivity and quality.

In the present study, we focused on understanding endogenous cAMP and phytohormone interactions, the relevant morphological effects and molecular regulatory mechanisms, through developing an inducible *AC* transgenic model of *Brassica napus* (also known as rapeseed, rape, oilseed rape), an important oilseed crop worldwide. The results have advanced our knowledge of cAMP signaling in higher plants and offered the prospect of its potentially practical application in crop improvement.

## 2. Materials and methods

### 2.1. Plant materials and growth conditions

Allotetraploid *Brassica napus* cv. P300, a semi-winter homozygous inbred line derived from the cross “shuangyou9722 × 220”, was used to generate the transgenic plants in the present study. The P300 seeds were obtained from the Institute of Industrial Crops, Henan Academy of Agricultural Sciences, Zhengzhou, China. Plants were grown on potting mix (Pindstrup Mosebrug A/S, Ryomgaard, Denmark) in a growth room of 16 h light/8 h dark and 22°C.

### 2.2. Preparation of culture media and stock solutions

Full-strength Murashige and Skoog (MS) medium, 2-(N-morpholino)ethanesulfonic acid (MES), sucrose, agar, and Timentin were all purchased from Coolaber Technologies (Beijing, China). The stock solution of 1000 × Gamborg’s B5 vitamins was obtained from PhytoTech Labs (Lenexa, KS, USA). 6-Benzylaminopurine (BA), kinetin, zeatin, 2,4-D, 1-naphthalene acetic acid (NAA), silver nitrate (AgNO_3_), and hygromycin were all purchased from Sigma-Aldrich (St. Louis, MO, USA). To prepare stock solutions of 2,4-D (200 mg · L^−1^), NAA (200 mg · L^−1^), BA (200 mg · L^−1^) and kinetin (200 mg · L^−1^), the chemicals were first completely dissolved in a small amount of solvent (0.1 *N* HCl for BA and kinetin; ethanol for NAA and 2,4-D) and then brought to the final volume with deionized water; zeatin (5 g · L^−1^) and AgNO_3_ (3 g · L^−1^) were directly dissolved in deionized water. All the prepared stock solutions were filter-sterilized using a 0.22 µm filtration unit and aliquoted for storage in a refrigerator at −20°C, except that AgNO_3_ was stored at 4°C in an amber bottle.

For the preparation of culture media, mixed MS powder with deionized water, added an appropriate amount of sucrose, and adjusted the pH to 5.7 with KOH prior to the addition of agar. After sterilization by autoclaving for 20 min at 121°C and 1 kg · cm^−2^ (15 psi), allowed the medium to cool down to around 55°C, added the stock solutions of Gamborg’s B5 vitamins, AgNO_3_, and plant growth regulators to achieve the final desired concentration in the medium, mixed, and immediately poured the medium into culture vessels in a sterile environment.

### 2.3. Plasmid construction and *Agrobacterium* preparation

The binary plasmid pTA7001-AC has been described previously,^10^ which contains the N-terminal cDNA fragment of AtKUP7 coding for the soluble adenylate cyclase activity in a glucocorticoid-mediated transcriptional induction system. pTA7001-AC was introduced into *Agrobacterium tumefaciens* GV3101 and stored at −80°C. When used for genetic transformation, the *Agrobacterium* harboring pTA7001-AC was inoculated into 2 ml YEP liquid medium (10 g/L yeast extract +10 g/L Bacto peptone +5 g NaCl, pH 7.0) with the addition of antibiotics (50 mg/L kanamycin, 25 mg/L rifamycin, and 50 mg/L gentamycin), cultured with shaking (200 rpm) at 28°C for approximately 36–48 h, and then continued for 12 h after transferring into fresh the same medium in the ratio of 1:100. The bacterial pellet was harvested by centrifugation at 4000 rpm for 10 min, rinsed and resuspended with liquid infection medium (MS basal salt +30 g/L sucrose +100 µM acetosyringone) to approximately 0.4 of OD_600_, which was kept at 4°C for infection of rapeseed explants.

### 2.4. *Agrobacterium tumefaciens*‑mediated genetic transformation

*Agrobacterium*-mediated genetic transformation of hypocotyls in rapeseed was performed using a modified procedure of the previous report,^35^ wherein MSB5 basal medium (MS basal salt + Gamborg’s B5 vitamins + 500 mg/L MES + 30 g/L sucrose + 7 g/L agar, pH 5.7) was used. Specifically, the seeds of *B. napus* cv. P300 were surface sterilized with 70% ethanol for 30 s, followed by 0.1% (w/v) HgCl_2_ for 5 min, and then rinsed 3 ~ 5 times with sterile ultrapure water. The seeds were planted on a germination medium (GM; 1/2 MS basal salt + 500 mg/L MES + 10 g/L sucrose + 7 g/L agar, pH 5.7) to grow for approximately 5 days under condition of light shading by black cloth in a growth chamber that was maintained with the photoperiod of 16 h day/8 h night at a temperature of 22°C to 20°C (day/night), relative humidity of 65%, and light intensity of 5000 lux with cool-white fluorescent lamps. The hypocotyls of five-day-old dark-grown seedlings were excised and cut to 0.5 ~ 1 cm segments in length, placed on a pre-culture medium (PCM; MSB5 + 1 mg/L 2,4-D + 2 mg/L BA + 0.05 mg/L NAA) to incubate for 3 days under dark condition in the growth chamber above and then transferred into a sterile plate containing the aforementioned *Agrobacterium* suspension to infect for approximately 10 min. Next, the infected hypocotyls was placed on cocultivation medium (PCM +100 µM acetosyringone) for 3 days, followed by transferring the hypocotyls to calli-inducing medium (CIM; MSB5 + 1 mg/L 2,4-D + 1 mg/L kinetin + 5 mg/L AgNO_3_ + 300 mg/L Timentin + 4 mg/L hygromycin) for 7 days under dark condition in the growth chamber. Subsequently, transferred the hypocotyls with embryogenic calli into a shoot-inducing medium (SIM; MSB5 + 1 mg/L zeatin + 3 mg/L BA + 5 mg/L AgNO_3_ + 300 mg/L Timentin + 4 mg/L hygromycin) for the regeneration of shoots, and renewed the medium every 2 weeks. After 6 ~ 8 weeks, the hypocotyls with green buds were transferred into the bud extension medium (BEM; MSB5 + 0.5 mg/L BA + 5 mg/L AgNO_3_ + 300 mg/L Timentin). When growing at least three leaves, the induced shoots were transferred into a root-inducing medium (RIM; GM + 0.1 mg/L NAA). Finally, the rooted plantlets were transplanted to pots with nutritive soil after acclimatization, vernalized in a lighting incubator at 4°C for approximately 4 ~ 6 weeks, and subsequently cultivated to flower and set seeds in the growth room. In the aforementioned media, the antibiotic Timentin was added for the elimination of *Agrobacterium*, and hygromycin was used for the selection of transformants; acetosyringone was included to enhance *Agrobacterium* virulence.

### 2.5. PCR‑based identification of transgenic rapeseed plants

Genomic DNA was isolated from young leaves of putative transformants (T_0_) using AmPure Plant DNA Kit (Magen Biotechnology, Guangzhou, China), and used for PCR amplification to determine the integration of T-DNA in the genome. PCR amplification was performed on a T100 thermal cycler (Bio-Rad, Hercules, CA, USA). A total of 20 µL per PCR reaction mixture was prepared using 2×Es Taq MasterMix (CoWin Biosciences, Cambridge, MA, USA) and the gene-specific primers (Table S1) for amplification of a 595 bp fragment of the hygromycin-resistance gene and a 315 bp fragment of the *AC* transgene. PCR conditions consisted of an initial hold at 94°C for 2 min; followed by 30 cycles of 94°C for 30 s, 52°C or 57°C for 30 s, 72°C for 30 s; and finally terminated with a step at 72°C for 5 min. Amplification products were analyzed by electrophoresis in 1.5% agarose gel with ethidium bromide staining and photographed under UV light.

### 2.6. DEX treatment

Dexamethasone (DEX), a glucocorticoid derivative, was purchased from Sigma-Aldrich. DEX was dissolved in DMSO to make a stock solution of 30 mM before use. Plants grown on potting mix were thoroughly sprayed with a water solution containing 30 µM DEX with the addition of 0.01% Tween-20 as a wetting agent, and then covered overnight using a transparent plastic dome before returning to regular growing conditions.

### 2.7. Determination of cAMP and phytohormone contents

Four-week-old plants were subjected to DEX treatment, and aerial part tissue samples were collected at the indicated time points after DEX spray. The contents of cAMP and phytohormones were determined as previously described.^10^ Briefly, tissue samples were homogenized in liquid nitrogen, extracted with 1 M HClO_4_, and cAMP was quantified with three biological replicates using a commercial cAMP ELISA Detection Kit (GenScript, Nanjing, China). For the measurement of phytohormones, homogenized tissue samples were extracted with methanol, and analyzed in triplicate using the AB SCIEX Q-TRAP® 6500 LC-MS/MS system (Applied Biosystems, Waltham, MA, USA).

### 2.8. Quantitative RT-PCR

True leaf tissue samples were collected from four-week-old seedlings at the indicated time points after DEX spray and used for detecting transcripts of *AC* transgene. For all other genes, aerial part tissue (excluding cotyledons) samples from five-week-old seedlings were used. Total RNA was isolated using the Spin Column Plant Total RNA Purification Kit (Sangon Biotech, Shanghai, China). After *DNase I* digestion, total RNA was subjected to reverse transcription (RT) using the HIScript® III RT SuperMix for qPCR (+ gDNA wiper) Reagent Kit (Vazyme Biotech, Nanjing, China). The ChamQ^TM^ Universal SYBR® qPCR Master Mix (Vazyme Biotech) was used to prepare PCR reaction mixture for amplification on LightCycler® 480 II Real-Time PCR System (Roche, Basel, Switzerland) with the following conditions: 30 s at 95°C, 40 cycles of 95°C for 10 s, and 60°C for 30 s. All primers used in this study are listed in Table S1. Melting curve analysis and agarose gel electrophoresis were performed to verify the specificity of amplified products. Relative expression levels were normalized to an internal control gene *F-box* and calculated using the 2^–∆∆Cq^ method. *F-box* has been verified by systemic studies in *Brassica napus* to serve as an optimal reference gene for quantitative RT-PCR normalization in a previous publication.^36^ In addition, independent amplification experiments with *F-box* showed its stable expression in the samples under control conditions and DEX treatment, which further validated the suitability of *F-box* for the analyses (Table S2). Three biological replicates were analyzed in all these experiments.

The sequences of *Brassica napus* genes were obtained from the Bra_napus_v2.0 genome assembly of cultivar ZS11 (https://www.ncbi.nlm.nih.gov/assembly/GCF_000686985.2/).

### 2.9. Phenotypic measurements

For each genotype, 10 healthy seeds were sown in a potting mix. After 4 weeks, the growing seedlings were treated with DEX spray as described above. Growth characteristics of five-week-old plants were determined by the following parameters: plant height (from the cotyledon node to the shoot tip), hypocotyl length (from the cotyledon node to the root base), maximum of root length (from the base to tip of root system), leaf length (from the base to tip of the blade), leaf or cotyledon width (the widest portion of the blade), the leaf width/length ratio representing leaf shape, leaf or cotyledon petiole length, the 1^st^ internode length above the cotyledons (distance from the cotyledon node to the 1^st^ true leaf node), and fresh shoot biomass (the aboveground fresh weight). Seed diameter was measured at the widest portion and calculated on average from 10 mature seeds. 1000-seed weight was estimated on average from weighing 100 mature seeds in triplicate. All the above morphometric measurements were made using a caliper.

### 2.10. Transcriptome sequencing and bioinformatic analysis

About four-week-old AC#3 transgenic plants were subjected to DEX treatment as described above. Whole plant tissue samples were collected at 0 h and 24 h after DEX treatment, each with three biological replicates and each replicate containing five individual plants. All the samples were snap-frozen in liquid nitrogen, and total RNA was isolated using the Spin Column Plant Total RNA Purification Kit (Sangon Biotech). All the downstream analyses were provided by Biomarker Technologies (Beijing, China). RNA concentration and purity were measured using NanoDrop™ 2000 Spectrophotometer (Thermo Fisher Scientific, Waltham, MA, USA), and RNA integrity was assessed using Agilent Bioanalyzer 2100 system (Agilent Technologies, Santa Clara, CA, USA). Sequencing libraries were prepared using NEBNext® Ultra™ RNA Library Prep Kit for Illumina (NEB, Ipswich, MA, USA) following the manufacturer’s recommendations. The library preparations were sequenced on an Illumina platform, and paired-end reads were generated. Raw data were processed to obtain clean reads by removing adapter sequences and low-quality sequence reads. Then, clean reads were mapped with the Bra_napus_v2.0 genome assembly of cultivar ZS11 (https://www.ncbi.nlm.nih.gov/assembly/GCF_000686985.2/) as reference genome using the HISAT2 program.^37^ Only reads with a perfect match or one mismatch were further analyzed and annotated based on the reference genome. Gene function was annotated based on public databases. Gene expression levels were estimated by fragments per kilobase of transcript per million fragments mapped (FPKM). Differential gene expression analysis was performed using the DESeq2 package.^38^ The resulting *p* values were adjusted using the Benjamini–Hochberg procedure for controlling the false discovery rate (FDR). Differential expression genes (DEGs) were determined at the threshold of an absolute value of log2 (fold change) > 1 and FDR < 0.05.

KEGG (Kyoto Encyclopedia of Genes and Genomes) pathway enrichment analysis was conducted using KOBAS.^39^ GO (Gene Ontology) enrichment analysis was implemented by the GOseq R packages based on Wallenius non-central hypergeometric distribution,^40^ which can adjust for gene length bias in DEGs.

### 2.11. Statistics

The statistical software package GraphPad Prism version 8.0.2 (GraphPad Software, San Diego, CA, USA) and Microsoft Excel program (Microsoft, Redmond, WA, USA) were used for data analysis.

## 3. Results and discussion

### 3.1. Generation of AC transgenic plants in rapeseed

**Figure 1.**
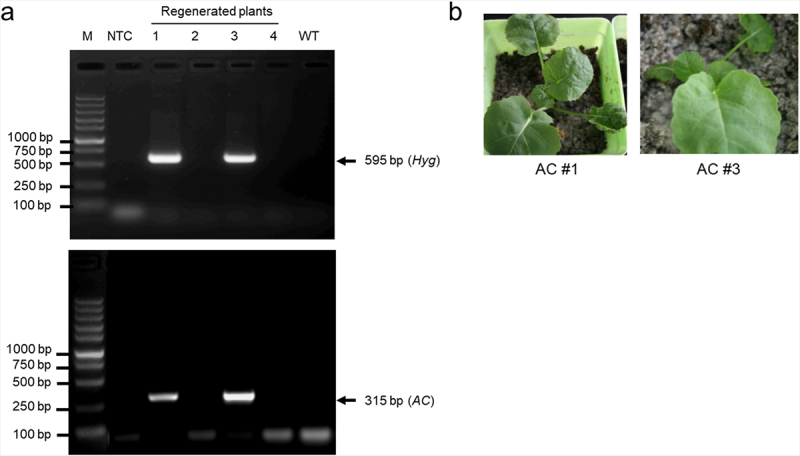
Generation of *AC* transgenic rapeseed plants. Rapeseed hypocotyl explants were used for tissue culture and *Agrobacterium*-mediated genetic transformation of the binary plasmid pTA7001-AC. (a) PCR-based verification of the regenerated plants. The DNA fragments of 595 bp from the hygromycin resistance gene (*Hyg*; upper panel) and 315 bp from the *AC* transgene (*AC*; bottom panel) in the T-DNA region of pTA7001-AC were specifically amplified in the regenerated #1 and #3 plants, while no amplification in the regenerated #2 and #4 plants, wild type (WT) and a non-template control (NTC) reaction. M, DNA size marker; (b) *AC* transgenic rapeseed seedlings. The verified #1 and #3 transgenic plants in (a) were referred to as AC#1 and AC#3, respectively.

A binary plasmid pTA7001-AC has been constructed to code for the soluble AC activity and confirmed to confer cAMP production in *Arabidopsis thaliana* transgenic plants in our previous study.^10^ To verify its applicability in a different species, we introduced pTA7001-AC into rapeseed via tissue culture of hypocotyl explants and *Agrobacterium*-mediated genetic transformation. Four regenerated plantlets were obtained, and subsequently analyzed by PCR verification using genomic DNA as the template and gene-specific primers (Table S1) for the hygromycin resistance gene and *AC* transgene in the T-DNA region of pTA7001-AC. Two of the regenerated plantlets (#1 and #3) each showed the specific amplification of a 595 bp DNA fragment from the hygromycin resistance gene (Figure 1a, upper panel) and a 315 bp DNA fragment from the *AC* transgene (Figure 1a, bottom panel), while no PCR product was amplified from the other regenerated plantlets (#2 and #4) as well as either wild type (WT) plants or a non-template control (NTC) reaction. Thus, we confirmed two independent *AC* transgenic lines, hereafter referred to as AC#1 and AC#3 (Figure 1b).

### 3.2. Inducible expression of AC transgene and simultaneous elevation of cellular cAMP in rapeseed

**Figure 2.**
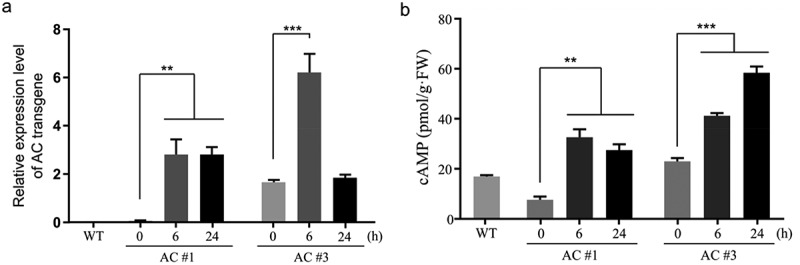
Inducible expression of *AC* transgene and simultaneous cAMP elevation in rapeseed. (a) Relative levels of *AC* transcripts. Quantitative RT-PCR was performed to determine the relative expression levels by normalization to *F-box* gene using the 2^–∆∆Cq^ method. *AC* transcripts were undetectable in wild-type (WT) plants. AC#1 and AC#3 represent two independent transgenic lines; (b) cellular cAMP contents. Quantification of cAMP was conducted by the enzyme-linked immunosorbent assay (ELISA). True leaf (a) or aerial part (b) tissue samples of four-week-old seedlings were collected at the time points of 0, 6, and 24 h after dexamethasone (DEX) spray. Data are presented as the mean ± SD (*n* = 3); two-tailed Student’s *t*-test, ** *p* <.01, *** *p* <.001.

The *AC* transgene was engineered under the control of a steroid-inducible expression system.^41^ To induce its expression, four-week-old seedlings of AC#1 and AC#3 transgenic lines were thoroughly sprayed with a water solution containing 30 μM dexamethasone (DEX, a strong synthetic glucocorticoid) plus 0.01% Tween-20 as a wetting agent.^10^ Subsequently, true leaf samples were collected at 0, 6, and 24 h after DEX spray and detected by quantitative RT-PCR using gene-specific primers (Table S1). The results showed that *AC* transcripts were significantly induced at 6 h, compared to that at 0 h representing the basal level, in both AC#1 and AC#3 transgenic lines (Figure 2a). It was noted that the *AC* transcripts were also detected with a significant level at 0 h in both AC#1 (Student’s *t*-test, *p* <.01) and AC#3 (*p* <.001) transgenic lines, while being not present in WT plants, indicating the occurrence of *AC* leaky expression which is a commonly observed phenomenon with chemical-inducible transgenic systems.^42^ AC#1 transgenic line showed a similar level of *AC* transcripts at 6 and 24 h after DEX spray; however, AC#3 transgenic line had a much higher level (2.2-fold) at 6 h than that in AC#1 and dramatically declined at 24 h reaching to the basal level (0 h) (Figure 2a), indicating that *AC* transcription in this line may be subjected to an impact by the autoregulatory negative feedback control mechanism, a typical feature of cAMP signaling.^10^

In all living organisms, AC activity is the sole source to synthesize cAMP (3’,5’-cAMP) and rapidly increase intracellular cAMP levels.^5^ Thus, we examined the levels of cAMP in tissue samples from the aerial part collected at 0, 6, and 24 h after DEX spray in four-week-old *AC* transgenic seedlings. The results indicated that cAMP was significantly increased from 7.66 pmol/g·FW (fresh weight) at 0 h to 32.53 pmol/g·FW at 6 h and 27.41 pmol/g·FW at 24 h after DEX spray in AC#1 transgenic line, and it was significantly elevated from 22.98 pmol/g·FW at 0 h to 41.13 pmol/g·FW at 6 h and 58.38 pmol/g·FW at 24 h after DEX spray in AC#3 transgenic line; in contrast, it was 16.88 pmol/g·FW in WT plants (Figure 2b). Thus, we confirmed an inducible activity of the *AC* transgene to synthesize cAMP in the transgenic rapeseed plants. It was noted that the abundance of *AC* transcripts was not well correlated with the level of cAMP content at 24 h after DEX spray in AC#3 transgenic line, which might reflect the result of an autoregulatory feedback control mechanism of cAMP signaling in this line, although we cannot rule out effects by the complex regulatory mechanisms of mRNA decay, protein turnover, posttranslational modifications, and metabolic adaptation during the processes of gene expression pathway and metabolic reactions. This finding suggested that the transient elevation of *AC* transcripts in plants might be enough to rapidly produce enzymatic activities and cAMP; thus, cellular cAMP contents might change dynamically and represent the endpoint measurements. Recently, it was shown that pathogen infection stimulated a significant elevation of intracellular cAMP at 4 h and further increase at 24 h after inoculation in Arabidopsis plants.^43^ Cellular cAMP elevation triggers a series of downstream signaling events.^12,44^ Thus, we concluded that the above-generated inducible *AC* transgenic lines may serve as a genetic model system for mimicking the stimulation of cAMP signaling in nature.

### 3.3. Endogenous cAMP elevation disturbs IAA and stress-hormone homeostasis in rapeseed

**Figure 3.**
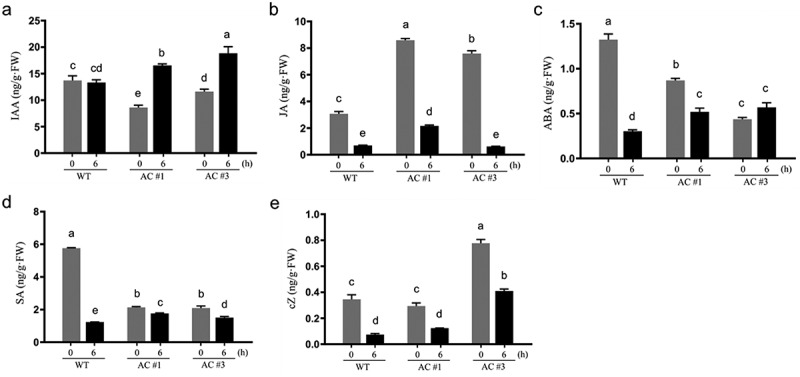
Effects of endogenous cAMP elevation on phytohormone contents in rapeseed. Aerial part tissue samples of four-week-old seedlings were collected at the time points of 0 h and 6 h after dexamethasone (DEX) spray in AC#1 and AC#3 transgenic lines along with wild type (WT). Quantification of phytohormones was performed by liquid chromatography-tandem mass spectrometry (LC-MS/MS) in triplicates. (a) Indole-3-acetic acid (IAA); (b) jasmonic acid (JA); (c) abscisic acid (ABA); (d) salicylic acid (SA); (e) *cis*-zeatin (cZ). Note: DEX itself affects the contents of different phytohormones except IAA, as seen between 6 h versus 0 h in WT plants. The significant changes of basal levels (0 h) in the *AC* transgenic lines compared to WT may be best explained by the impacts of constitutive *AC* leakiness expression during the growth period of transgenic plants, and thus represent the result of long-term continuous cAMP elevation stimulation; in contrast, the significant changes of IAA levels specifically induced by DEX in the transgenic lines may represent the result of short-term or acute cAMP elevation stimulation. Data are presented as the mean ± SD (*n* = 3), different letters on the bars indicating statistical significance at *p* <.05 by Tukey’s multiple comparisons test following two-way ANOVA.

We have recently noted that cellular cAMP elevation resulted in altered phytohormone contents in *Arabidopsis*.^10^ To verify this observation in a different species, we measured various phytohormones in the aerial part tissues of four-week-old seedlings at 0 h and 6 h after DEX spray in rapeseed. Several classes of phytohormones, including gibberellic acid (GA_3_), gibberellin A4 (GA_4_), kinetin (6-KT), and brassinosteroid (BR), were undetectable in the samples. Surprisingly, it was noted that salicylic acid (SA), jasmonic acid (JA), abscisic acid (ABA), and *cis*-zeatin (cZ) contents were significantly reduced between 6 h versus 0 h after DEX spray in WT plants (Figure 3), suggesting that DEX treatment by itself had an impact on these phytohormones. DEX is a synthetic steroid and has been widely used in glucocorticoid-mediated inducible expression systems for gene function analysis in transgenic plants over the past decades, including those genes (e.g., *IPT*, *IAA1*, *SUPERMAN*) involving hormonal biosynthesis and regulation.^10,41,45–50^ However, previous reports have rarely noted the effect of DEX alone on endogenous phytohormones in plants until now. It has been documented that glucocorticoid-mediated transcriptional induction systems may cause phenotypic growth defects in tobacco and *Arabidopsis* under conditions of high concentration and/or prolonged DEX treatments.^51,52^ In the present study, we did not observe any phenotypic growth defect in either WT or *AC* transgenic plants under the DEX treatment regime, which may rule out the possibility of DEX overdose. In agreement with this notion, DEX treatment showed no impact on IAA contents in WT plants at all; however, IAA contents were significantly increased by 1.9-fold and 1.6-fold on average between 6 h versus 0 h after DEX spray in AC#1 and AC#3 transgenic lines, respectively (Figure 3a). When compared to WT plants, IAA contents at 6 h after DEX spray were induced to significantly increase by 1.2-fold and 1.4-fold in AC#1 and AC#3 transgenic lines, respectively. These data indicated a specific effect by the DEX-induced cAMP elevation that significantly enhances IAA levels in the *AC* transgenic plants. Early studies had shown that exogenous cAMP application mimicked the action of IAA in activating the *de novo* synthesis of tryptophan oxygenase in chickpea (*Cicer arietinum*),^53^ whereas cAMP and auxin synergistically enhanced cell elongation in sunchoke (*Helianthus tuberosus*) tuber slices.^54^ Recently, TIR1/AFB auxin receptors have been reported to possess AC activities which are stimulated by auxin to generate cAMP playing an important function toward auxin signaling.^28^ Cyclic nucleotide-gated ion channels (CNGCs) are among the known direct binding targets of cAMP, and CNGC2 was shown to implicate the regulation of auxin biosynthesis in *Arabidopsis*.^55^ CNGCs are highly conserved during evolution but constitute a much larger family in plants than animals.^56^ While they mainly mediate Ca^2+^ flux and signaling, CNGCs affect various aspects of plant growth and development.^57^ In animals, sAC is directly activated by Ca^2+^ and bicarbonate and acts as a sensor for Ca^2+^, bicarbonate/CO_2_/pH, and ATP at various intracellular locations^13^; it links Ca^2+^ influx to cAMP generation,^58^ making Ca^2+^-induced AC activity the link between CNGCs and cAMP. Thus, CNGCs may serve as hubs for interactions between Ca^2+^, cAMP, and phytohormones in plants.

Due to the unexpected impacts of DEX itself on JA, ABA, SA, and cZ contents (Figure 3b–e), it became difficult to evaluate the specific effects of DEX-induced cAMP elevation on these phytohormones. However, *AC* transgene showed notable leaky expression in the transgenic plants (Figure 2a), and thus we were able to compare the basal levels of phytohormones as measured at 0 h after DEX spray in these plants. In comparison with WT plants, JA basal levels in both AC#1 and AC#3 transgenic lines significantly increased by 2.8- and 2.5-fold on average, respectively (Figure 3b); ABA basal levels in both AC#1 and AC#3 decreased by 0.7- and 0.3-fold, respectively (Figure 3c); SA basal levels of both AC#1 and AC#3 were reduced by 0.4-fold (Figure 3d). cZ basal levels showed no obvious change (0.9-fold) in AC#1 compared to WT, but they significantly increased by 2.3-fold in AC#3 (Figure 3e). Differences in the basal levels of phytohormones between the *AC* transgenic plants and WT may be best explained by the impact of *AC* leakiness expression, and thus represent the result of long-term continuous cAMP elevation stimulation. While the significant elevation of IAA contents between 6 h versus 0 h after DEX spray in the transgenic plants (Figure 3a) may represent the result of short-term or acute cAMP elevation stimulation, it should be reasonable to expect an observable effect of continuous cAMP stimulation on IAA. Indeed, IAA basal levels were significantly altered in both AC#1 and AC#3 transgenic plants compared to WT (Figure 3a), albeit in the opposite direction to the acute effect. It has long been known that phytohormone contents display dynamic changes in plants,^59^ and thus the results (Figure 3) represent endpoint measurements. The observed reduction of IAA basal levels by continuous cAMP stimulation (Figure 3a), in contrast with the rapid IAA elevation by acute cAMP stimulation (Figure 3a), was likely caused by the autoregulatory negative feedback control mechanism of phytohormones,^29,60^ due to the continuous cAMP stimulation. Nevertheless, the above results supported that endogenous cAMP elevation not only conferred significant impacts on IAA but also JA, ABA, and SA. Interestingly, JA has been reported to induce rapid cAMP elevation in Arabidopsis.^61^ 8-Br-cAMP, a membrane-permeable cAMP analog, was found to completely reverse exogenous ABA-induced inhibition of stomatal opening,^62^ while ABA inhibited cAMP-induced seed germination of lettuce (*Lactuca sativa*).^24^ In an ABA-deficient mutant of maize, it was demonstrated that exogenous ABA application regulated endogenous cAMP increase caused by drought and heat stresses, and modulated cAMP-mediated protein expression^63^; moreover, the AC activity of ZmRPP13-LK3 protein was implicated in ABA-regulated heat resistance in maize.^22^ Both 8-Br-cAMP and forskolin (a specific AC activator) significantly stimulated SA levels, whereas the AC inhibitor DDA (2’,5’-dideoxyadenosine) dramatically reduced the toxin-induced SA increases in *Arabidopsis*.^64^ Overall, all the above findings supported the interplays between cAMP and stress hormones JA, ABA, and SA.

Phytohormones function as a network by auto-regulating themselves and cross-regulating each other by acting as negative feedback regulators in plants.^29,65,66^ Thus, cAMP and phytohormones may feature very complex *in planta* interactions being operated at the network level, which will depend on the temporal and spatial regulations in different cells or tissue types in different plant species. Indeed, cAMP elevation significantly altered IAA levels in rapeseed (Figure 3a), but such an impact was not observed in *Arabidopsis* previously.^10^ Based on the results representing the endpoint measurements (e.g., Figure 3) of phytohormones in a complex signaling network, it might not warrant completely ruling out the inherent connection of cAMP with other phytohormones. GA_3_ modulated cAMP levels during seed germination of *Phacelia tanacetifolia*,^23^ and the combination of exogenous cAMP with a low concentration of GA_3_ promoted lettuce seed germination.^24^ Interestingly, 8-Br-cAMP used in combination with IAA was able to replace the cell division-promoting activity of cytokinin in plant tissue culture,^27,67^ indicating the intrinsic relations among cAMP, IAA, and cytokinin. Altogether, we supposed that cAMP may be interconnected with various phytohormones by the mechanisms of mutual regulation in an intricate and ingenious system.

### 3.4. Endogenous cAMP elevation modulates IAA biosynthesis and stress-hormone signaling signature genes in rapeseed

**Figure 4.**
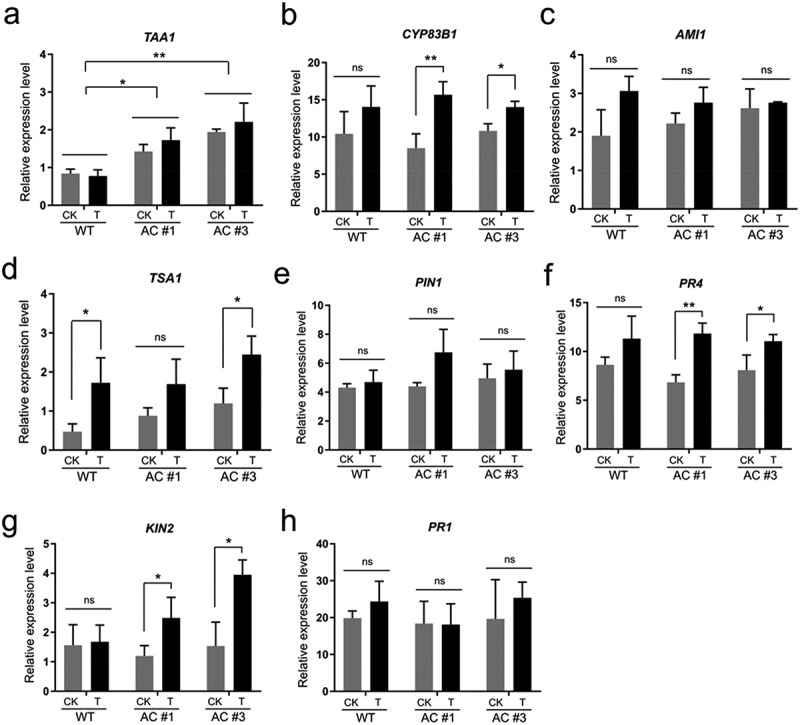
Endogenous cAMP elevation modulates the expression of IAA biosynthesis and stress-hormone signaling signature genes. Aerial part tissue samples of five-week-old seedlings collected at the time point of 0 h (CK) and 6 h (T) after dexamethasone (DEX) treatment in both AC#1 and AC#3 transgenic lines, along with wild type (WT), were detected by quantitative RT-PCR with normalization to *F-box* gene. (a) *TAA1* (tryptophan aminotransferase of Arabidopsis 1); (b) *CYP83B1* (cytochrome P450 83B1); (c) *AMI1* (amidase 1); (d) *TSA1* (tryptophan synthase alpha subunit); (e) *PIN1* (PIN-FORMED 1); (f) *PR4* (pathogenesis-related protein 4); (g) *KIN2* (stress-induced protein KIN2); (h) *PR1* (pathogenesis-related protein 1). Data are presented as the mean ± SD of three biological replicates. Two-tailed Student’s *t*-test, *p <.05, **p <.01.

To gain insight into the mechanisms of cAMP elevation to mediate phytohormone changes (Figure 3), we examined a set of signature genes (Table S1) in relevance to IAA biosynthesis and stress-hormone signaling pathways using quantitative RT-PCR. Currently, several pathways for IAA biosynthesis in plants have been documented, including the tryptophan (Trp)-independent pathway and four Trp-dependent pathways, i.e., the routes of indole-3-acetamide (IAM), indole-3-pyruvic acid (IPA), tryptamine (TAM), and indole-3-acetaldoxime (IAOx).^68,69^ Detection of *TAA1*, a gene encoding tryptophan aminotransferase that converts Trp into IPA in the IPA pathway, showed no significant expression changes between 6 h versus 0 h after DEX spray in both AC#1 and AC#3 transgenic lines; however, it was noted that the basal levels (0 h) of *TAA1* expression significantly increased in all the transgenic lines compared to WT (Figure 4a), indicating the effect of continuous *AC* leakiness expression and cAMP production. We postulated that the highly elevated basal levels (1.7-fold in AC#1; 2.3-fold in AC#3) of *TAA1* in the transgenic plants than WT might limit its further response to DEX induction through the autoregulatory negative feedback mechanism of phytohormone signaling.^60,66,70,71^ This notion agreed with the result that both AC#1 and AC#3 transgenic lines displayed significantly reduced IAA basal levels due to the feedback regulation (Figure 3a). The IAOx pathway is closely related to quite a few members of the cytochrome P450 (CYP) superfamily, e.g., CYP79B2, CYP79B3, CYP71A13, and CYP83B1.^72,73^ Of them, *CYP83B1* is significantly induced by exogenously applied IAA in *Arabidopsis* seedlings,^74^ and it modulates IAA production by controlling the flux of IAOx into IAA and indole glucosinolate biosynthesis.^73^ We found that *CYP83B1* was significantly upregulated between 6 h versus 0 h after DEX treatment consistently in both AC#1 and AC#3 transgenic lines (Figure 4b), which was in line with the elevated IAA levels (Figure 3a). We did not detect significant expression changes of *AMI1* in the IAM pathway (Figure 4c). *TSA1* in the Trp-independent pathway showed a significant increase in AC#3 but not AC#1 transgenic line between 6 h versus 0 h after DEX treatment; moreover, a similar result was also observed with the basal levels of *TSA1* (Figure 4d), suggesting a nonspecific effect of cAMP elevation on *TSA1*. Additionally, we did not detect transcriptional changes in *PIN1* (Figure 4e), a key gene mediating polar auxin transport in developing plant tissues.^75^ Overall, the above results indicated that cAMP elevation stimulated gene expression of the IPA and IAOx pathways for IAA biosynthesis in rapeseed plants. The phytohormone auxin is predominantly represented by IAA and regulates many aspects of plant growth and development.^76^ The IPA pathway has been suggested to be the main IAA biosynthesis pathway in the plant kingdom, whereas the IAOx pathway is considered the Brassicaceae species-specific one.^68^ Thus, our above results suggested that cAMP signaling is crucial to the regulation of IAA-mediated growth and development in plants, which is well in line with the most recent findings that TIR1/AFB auxin receptors possess AC activity to produce cAMP acting as a second messenger in TIR1/AFB-mediated auxin perception and signal transduction in plants.^28^

JA, ABA, and SA form a defense crosstalk network against environmental stresses and play a crucial role in regulating signaling pathways.^77^ Given the significant impacts of cAMP elevation on the contents of these stress hormones (Figure 3), we examined whether stress-related marker genes were altered in *AC* transgenic plants. The results showed that *PR4* and *KIN2* were significantly upregulated between 6 h versus 0 h after DEX treatment consistently in both AC#1 and AC#3 transgenic lines, while no obvious changes in WT plants (Figure 4f, g); in contrast, *PR1* did not change in either *AC* transgenic plants or WT after DEX treatment (Figure 4h). *PR1* and *PR4* are considered the signature genes of SA and JA signaling activation in plants, respectively,^78^ while *KIN2* is induced by ABA treatment and represents a prominent marker gene of ABA signaling.^79,80^ Thus, *PR4* and *KIN2* upregulation (Figure 4f, g) may prove the activation of JA and ABA signaling pathways in *AC* transgenic plants. While we did not detect *PR1* expression changes (Figure 4h), it has been reported that JA signaling and SA signaling can compromise one another, and the result is that plant tissues usually can activate either SA or JA signaling, but not both.^59^ Activation of the above stress-hormone signaling pathways may involve their crosstalk with IAA changes. It has long been demonstrated that *TAA1*-mediated auxin biosynthesis is essential for hormone crosstalk and plant development.^81^ Recent advances indicated that crosstalk between auxin and ABA signaling is vital for germination and seedling development in rapeseed,^82^ while JA interacts with many other hormones, including auxin and ABA, to act as a core signal in the phytohormone signaling network.^83^ After all, it should be emphasized that the observed results (Figure 4) may represent the integration and final output of a complex signaling network involving both synergistic and antagonistic interactions of various phytohormones,^66^ and the interplays with cAMP which exhibits a biphasic signaling pattern.^10^ It will be a great challenge to unravel the mechanisms underpinning the complex interactions between cAMP and phytohormones in a whole plant.

### 3.5. AC transgenic plants display IAA overproduction phenotypes in rapeseed

**Figure 5.**
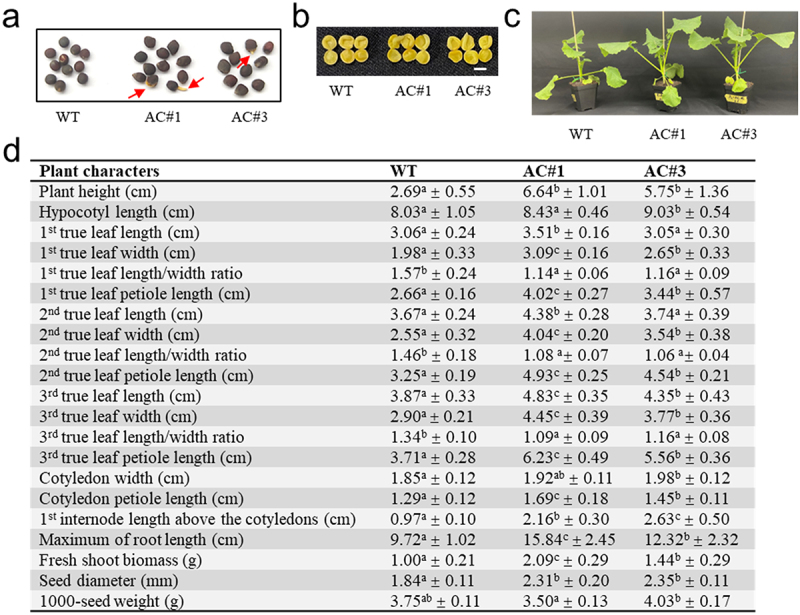
Phenotypic characteristics of *AC* transgenic plants in comparison with wild type. (a) Mature seeds. A notable percentage of seeds present testa rupture or radicle protrusion through the seed coat (indicated by arrows) in both AC#1 (22.9%) and AC#3 (7.8%) transgenic lines, but not in wild type (WT). Note that the shape of *AC* transgenic seeds is like a flattened irregular oval, whereas WT seeds are spherical; (b) mature seeds cut in half showing inside less accumulation of oil droplets (and protein bodies) but enlarged cotyledons in the *AC* transgenic lines, which may affect seed morphology. Scale bar = 0.2 cm; (c) visual representation of four-week-old plants; (d) measurements of plant characters. Values are means ± SD (*n* = 10, except *n* = 3 for the 1000-seed weight) by measuring five-week-old plants and mature seeds for each genotype. The mean values in the same row denoted by different letters indicate a significant difference (*p* <.05) between genotypes for the character, based on Tukey’s multiple range test after performing ANOVA.

IAA is the main auxin that affects almost every aspect of growth and development in plants.^76^ Perturbation of IAA and stress-hormone homeostasis (Figure 3) and misregulation of the phytohormone signaling network (Figure 4) could lead to phenotypic changes in *AC* transgenic plants. It was noted that a considerable percentage of mature seeds in both AC#1 (22.9%) and AC#3 (7.8%) transgenic lines exhibited testa rupture or radicle protrusion through the seed coat (Figure 5a), which are normally seen during seed germination after imbibition, but such a phenomenon was not observed in WT seeds. This result indicated that seed development, likely involving the control of seed dormancy, was affected in *AC* transgenic plants. Seed dormancy depends on auxin levels and requires the coordinated action of auxin and ABA signaling in *Arabidopsis*.^84^ Recently, it was shown that seed hormonal imbalance has a big impact on germination in rapeseed,^82,85^ while JA may specifically induce the expression of indole glucosinolate (GLS) biosynthesis genes and play a role in the seed dormancy of rapeseed via the indole GLS-linked auxin biosynthesis.^86^ Coincidently, *CYP83B1* was significantly upregulated in *AC* transgenic lines (Figure 4b).

The shape of *AC* transgenic seeds was like a flattened irregular oval, whereas WT seeds were spherical (Figure 5a). The size of *AC* transgenic seeds appeared larger than WT seeds, as evidenced by a significantly increased diameter, but there was no obvious difference in the 1000-seed weights between them (Figure 5d). Visual observation by cutting the seed in half showed less accumulation of oil droplets (and protein bodies) but enlarged cotyledons in *AC* transgenic seeds compared to WT (Figure 5b), which might account for the observed changes in seed appearance (Figure 5a). Auxin plays important roles in embryo, seed, and fruit development or patterning.^87,88^ Higher endogenous IAA levels may lead to enhanced post-mitotic cell expansion, also known as compensated cell enlargement (CCE), and larger cotyledons in plants.^89^

Increased IAA levels in auxin overproduction mutants usually cause some typical auxin excess phenotypes, e.g., elongated hypocotyls, epinastic leaves, and enhanced apical dominance.^73,74,90^ In comparison with WT, *AC* transgenic plants grew larger in size with significantly increased plant height, longer cotyledon/leaf petioles, widened blades of the leaves (Figure 5c), the lengthened internode, increased root length, and enhanced fresh shoot biomass in both AC#1 and AC#3 transgenic lines (Figure 5d). In addition, hypocotyl length and cotyledon width also increased. All these overgrowth characteristics of *AC* transgenic plants compared to WT might be best explained by the cAMP-mediated IAA hyperaccumulation (Figure 3a). Auxin is best known primarily for promoting cell growth and elongation of the plant at low concentrations, and it can direct morphogenesis of root, shoot, and seed.^76,91,92^ Auxin is mostly produced in the apical meristem of shoots, young leaves, and developing seeds, and acts in a dosage-dependent way.^93,94^ However, localized auxin biosynthesis also plays essential roles in many developmental processes such as embryogenesis, seedling growth, vascular patterning, phyllotaxis, and root development.^92^ In contrast to gradually reduced root growth and auxin levels in Arabidopsis *taa1/tar* mutants, elevated auxin levels and dramatically elongated seminal roots due to enhanced cell elongation were observed in a hypomorphic mutant (*Bdtar2l*^*hypo*^) of *TAR2-Like* gene in *Brachypodium distachyon*.^95^ An overaccumulation of auxin in the leaf primordia caused an increase in the leaf width,^96^ whereas loss-of-function mutants of the YUCCA family genes involving auxin biosynthesis resulted in significantly reduced leaf width.^97^ Overproduction of IAA in *YUC8*-overexpressing lines conferred enhanced shoot growth and stem elongation.^98^ All these previous findings may well corroborate the effects of IAA overproduction responsible for the increased length of shoots, stems, and roots, as well as widened leaves in *AC* transgenic plants (Figure 5d). However, we should point out that these phenotypic changes represent the combined effects of both continuous and acute cAMP elevation stimulations under our experimental regime. Similar phenotypic changes were observed in AC#1 and AC#3 transgenic lines (Figure 5), which showed lower and higher cAMP basal levels than WT plants (Figure 2a), respectively, these phenotypic effects should be attributed to transient elevation of AC activities and cAMP levels, rather than the magnitude of cAMP basal levels. However, different cAMP basal levels may affect the sensitivity in responding to cAMP elevation in plants, as seen from our results (Figure 5d). These observations might highlight a critical role of cAMP dynamic changes in buffering plant growth and development, likely contributing to plant plasticity. Leaves are major organs for photosynthesis in plants, and leaf morphology is critical to achieving effective plant architecture for yielding performance. An overall modification of plant architecture in *AC* transgenic lines may be of potential application in rapeseed improvement and production.

### 3.6. cAMP-mediated CRGs are most significantly enriched in the plant hormone signal transduction pathway

**Figure 6.**
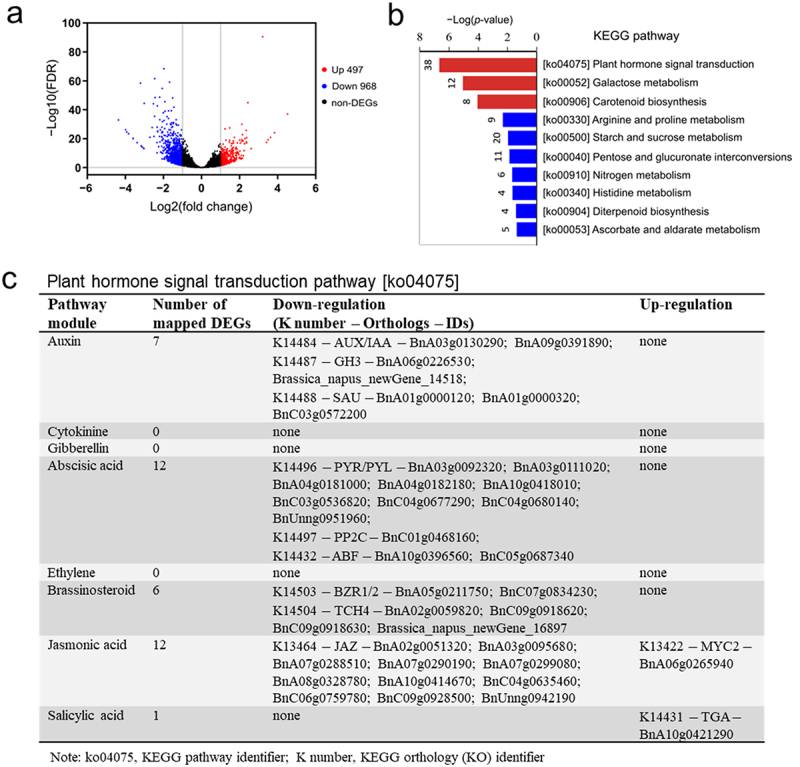
cAMP-mediated DEGs are most significantly enriched with plant hormone signal transduction pathway. Transcriptome sequencing analysis was performed with three biological replicates using whole plant tissue samples collected at 24 h (T) versus 0 h (CK) after dexamethasone (DEX) spray of four-week-old AC#3 transgenic plants. (a) Volcano plot showing differential expression genes (DEGs) of 497 up-regulation (red dots) and 968 down-regulation (blue dots). DEGs were determined using the estimated expression levels of FPKM (fragments per kilobase of transcript per million fragments mapped) at the threshold of an absolute value of log2(fold change) > 1 and a false discovery rate (FDR) < .05, and all of them are listed in table S3; (b) bar plot showing significantly enriched KEGG (Kyoto Encyclopedia of Genes and Genomes) pathways of DEGs at the cutoff of hypergeometric test *p* < .05 (table S4). Red bars indicate significant enrichment at FDR < .05. Shown on the top of each bar is the count of DEGs; (c) list of DEGs annotated in the plant hormone signal transduction pathway (figure S1). Gene IDs follow the Bra_napus_v2.0 genome assembly of cultivar ZS11 (https://www.ncbi.nlm.nih.gov/assembly/GCF_000686985.2/).

To better understand the regulatory roles of cAMP elevation at a genome-wide scale, we collected whole plant tissue samples and conducted transcriptome sequencing to identify differential expression genes (DEGs) between 24 h versus 0 h after DEX spray in AC#3 transgenic line. Here, AC#3 was used because it displayed a typical feature of cAMP signaling (i.e., transient and biphasic regulation; Figure 2),^10^ and a pattern of cAMP accumulation (Figure 2b) being similar to that stimulated by pathogens in plants.^43^ A total of 1465 DEGs were identified per the estimated expression levels of FPKM (fragments per kilobase of transcript per million fragments mapped) at the threshold of an absolute value of log2 (fold change) >1 and a false discovery rate (FDR) <0.05 (Figure 6a), and all of them are listed in Table S3. These DEGs represent a valuable resource of cAMP-responsive genes (CRGs) first identified in rapeseed, to the best of our knowledge. It is worth mentioning that these genes may respond to cAMP either directly or indirectly. For instance, an indirect response may be caused by Ca^2+^ signaling through the cAMP binding to cyclic nucleotide-gated channels (CNGCs).^9^ KEGG (Kyoto Encyclopedia of Genes and Genomes) enrichment analysis indicated that these DEGs were enriched in a total of 90 pathways (Table S4). Among them, 10 pathways were significantly enriched at the cutoff of the hypergeometric test *p* <.05, but only the top three were significant at the FDR of *q*-value <.05, i.e., plant hormone signal transduction, galactose metabolism, and carotenoid biosynthesis (Figure 6b, Table S4). In confirmation with the foregoing results (Figures 3 and 4), it was not surprising that plant hormone signal transduction was identified as the most significantly enriched pathway. A total of 38 DEGs were annotated with the plant hormone signal transduction pathway, while only 12 and 8 DEGs with galactose metabolism and carotenoid biosynthesis, respectively (Figure 6b). By looking over the KEGG pathway map of plant hormone signal transduction (Figure S1), we found that 7 DEGs were associated with the signaling cascade of auxin, 12 DEGs with ABA, 6 DEGs with BR, 12 DEGs with JA, and 1 DEG with SA; in contrast, no DEGs were identified with cytokinin (CK), gibberellin (GA), and ethylene (ET) (Figure 6c). This result was in line with the data of cAMP-mediated phytohormone content changes (Figure 3), providing additional evidence supporting the notion that the functions of cAMP are intrinsically associated with phytohormones and their signaling pathways. Consistently, we have previously demonstrated that plant hormone signal transduction was the most significantly enriched pathway with CRGs in *Arabidopsis*,^10^ suggesting evolutionary conservation in this respect.

### 3.7. cAMP-mediated CRGs are mainly enriched in functions relevant to hormonal, abiotic and biotic stress responses, as well as growth and development

**Figure 7.**
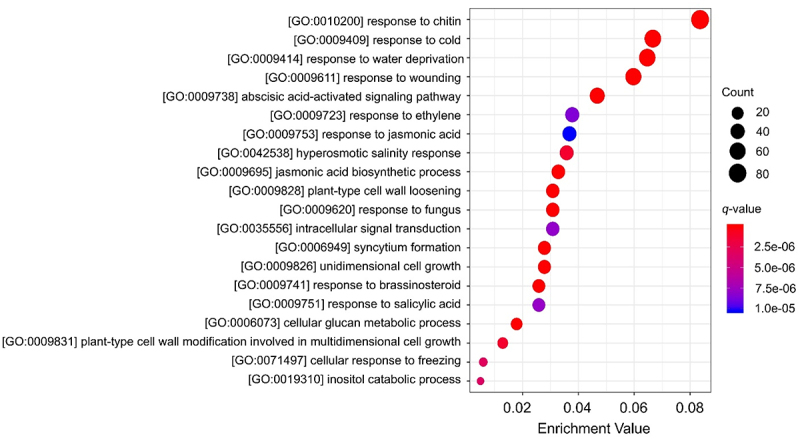
cAMP-mediated DEGs are mainly enriched in functions relevant to hormonal, abiotic and biotic stress responses, as well as growth and development. Gene ontology (GO) enrichment analysis was performed with the 1465 DEGs identified in Figure 6a, and the results are listed in table S5. Only the top 20 significantly enriched GO biological process terms are shown in the figure. The X-axis on the bottom indicates the enrichment value that represents the ratio of the enriched genes to the total number of genes in a certain GO biological process term in the database, while the Y-axis on the left indicates the enriched terms. The size and color of each dot represent the count of DEGs enriched in the indicated term and enrichment significance (*q*-value), respectively, both of which are illustrated by the scale legends on the right side of the chart.

All the above-identified DEGs (Figure 6a, Table S3) were subjected to Gene Ontology (GO) enrichment analysis, by which 1451 (99%) of them were functionally annotated in the database. Consequently, a total of 778 GO terms were enriched among the category of Biological Process (BP), 99 among Cellular Component (CC), and 394 among Molecular Function (MF) (Table S5). Per the cutoff of *q*-value <0.05, a total of 88 BP terms displayed significant enrichment, most of which were divided into three functional groups, i.e., hormonal (ABA, JA, SA, ET, auxin, GA, BR) responses or biosynthesis, abiotic stress (water deprivation, cold, wounding, salt, freezing, heat, mechanical stimuli, anoxia) and biotic stress or defense (fungus, chitin, respiratory burst, hypersensitive response, callose deposition, programmed cell death) responses, growth and development (unidimensional cell growth, cell morphogenesis involved in differentiation, post-embryonic development, transition from vegetative to reproductive phase, nodulation, sexual reproduction, seed germination, root development, fruit development, aging, stomatal movement, stamen filament development, cellulose microfibril organization), while the remaining BP terms were related to transcriptional regulation, nitrate signaling, starch and sucrose metabolism, etc. A profile for the top 20 significantly enriched BP terms is shown in Figure 7. Collectively, these results highlighted that cAMP plays prominent roles in regulating hormonal responses, abiotic and biotic stress responses, as well as growth and development, which agreed with our previous findings of CRGs in *Arabidopsis*,^10^ suggesting the conserved regulatory roles of cAMP in different species. Phytohormones are core regulators of plant growth and development, and they are also essential components for plants to balance stress and fitness.^99,100^ Based on all the above findings and previously accumulated knowledge, we suggested that activation of cAMP signaling by environmental and developmental stimuli may initiate extensive crosstalk with a variety of phytohormones by a complex signaling network for plants to optimize their growth and development. In this scenario, it should be rational to explain that plants need to maintain low levels of cellular cAMP to prevent any unfavorable overreaction of phytohormones, and that plant AC (and GC; guanylate cyclase) activities are embedded in moonlighting multifunctional proteins for facilitating the tight control of downstream signaling effects by producing an appropriate amount of cyclic nucleotides (cAMP and cGMP) within defined cytoplasmic areas or cellular compartments.^101^ Accordingly, temporally and spatially separated transient increases of cAMP production, together with phytohormones, may play critical roles in configuring the coordinated regulation of plant growth and development. It will be interesting to know if cAMP signaling might act to provide the molecular basis for orchestrating the action of different phytohormones in plants.

## 4. Conclusion

Recently, cAMP has been firmly confirmed to act as an essential signaling molecule in plants. However, our understanding of cAMP signaling in plants is very limited and greatly challenged by the moonlighting of adenylate cyclase activities embedded in multifunctional proteins, and by the low levels and transient elevation of cellular cAMP. In this study, we have introduced a DNA fragment encoding the adenylate cyclase catalytic center of AtKUP7 into rapeseed plants and confirmed an elevated cAMP production through induction of the transgenic expression in these plants. Thus, we were able to perform functional analyses by disturbing native cAMP signaling as a whole in rapeseed plants. We found that cAMP elevation results in altered levels of IAA and stress hormones (JA, ABA, and SA). Acute induction of cAMP production causes IAA overaccumulation and upregulation of IAA biosynthesis genes, as well as activation of ABA and JA signaling pathways. Typical overgrowth phenotypes related to IAA overdose were observed in these transgenic plants. We identified a set of 1465 CRGs which are most significantly enriched in plant hormone signal transduction pathway, and function mainly in relevance to hormonal, abiotic and biotic stress responses, as well as growth and development. Taken together, our results confirmed that cAMP elevation impacts phytohormone homeostasis and signaling, and modulates plant growth and development. We postulated that cAMP signaling may contribute to the coordinated regulation of growth and development in plants. Our understanding and genetic engineering of cAMP signaling may provide novel ideas and application perspectives in crop improvement.

## Supplementary Material

supplementary tables S3 to S5.xlsx

supplementary tables S1 and S2 and figure S1.docx

## Data Availability

All data generated or analyzed during this study are included in this article and its supplementary information files.
