# Supplementary material for: Endogenous cAMP elevation in Brassica napus causes changes in phytohormone levels
Source: Plant Signal Behav. 2024 Feb 5;19(1):2310963. doi: 10.1080/15592324.2024.2310963 (PMC10854363; doi:10.1080/15592324.2024.2310963)
Supplement: supplementary tables S1 and S2 and figure S1.docx [file KPSB_A_2310963_SM4381.docx]

**Table S1.** List of the gene-specific primers used in this study.

| Gene name | Sequence (5’ to 3’) | Size | Note^$^ |
| --- | --- | --- | --- |
| *AC* | Forward: ATGGCGGAGGAAAGCAGTAT  Reverse: TTATTTCCTCCCAACGGTCAAG | 315 bp | Adenylate cyclase transgene |
| *Hyg* | Forward: GCGAGAGCCTGACCTATTGCATCT  Reverse: GCCGTCAACCAAGCTCTGATAGAGT | 595 bp | Hygromycin |
| *AC* | Forward: GCAGGCTTTGACTTTCACCA  Reverse: CTCAGGTATCTCGTCCTCAT | 223 bp |  |
| *F-box* | Forward: GATAAGTCGCTTCCTACCGAAGT  Reverse: GTGTTCCCATTGCCCTGTATCAA | 183 bp | BnC03g0542750 |
| *TAA1* | Forward: TTGGTGTGTCTAAGGAGTCTCA  Reverse: GTTCTTCATCATCTCACGACCA | 116 bp | BnA02g0068470 |
| *CYP83B1* | Forward: CTTGACAATCTCACTGGTCTTAA  Reverse: GATCAATGAAACTCTCCGTCTCA | 133 bp | BnA08g0321230 |
| *AMI1* | Forward: AATGGTGTGTTGGTGATTCCGA  Reverse: GCTCACCTGACATAATCCAGAGA | 138 bp | BnA09g0389540 |
| *TSA1* | Forward: ATACCGTACATCACAGCTGGTGA  Reverse: ATGGGTCAGAGTAAGGAACTCCA | 115 bp | BnC09g0931770 |
| *PIN1* | Forward: TTCTGATCAAGCACCAAGGGTC  Reverse: GCTGCTTCTCTGGTAGTAGTCA | 88 bp | BnA06g0252060 |
| *PR4* | Forward: ACGATGTCAACTCCGTTCTCTTCA  Reverse: TTCATTCATCACAGGCTTGTTGTC | 128 bp | BnC05g0730310 |
| *KIN2* | Forward: GGTCGTGCTGAGGAGAAGT  Reverse: CTTGTTCATGCCGGTCTTCTC | 153 bp | BnA10g0412560 |
| *PR1* | Forward: GGAGAAAGTCTGTGAGAATTGG  Reverse: CCCGAGGATCATAGTTGCAAG | 84 bp | BnA03g0138420 |

^$^IDs of rapeseed homologous genes are sourced from the Bra_napus_v2.0 genome assembly of cultivar ZS11 (https://www.ncbi.nlm.nih.gov/assembly/GCF_000686985.2/)

**Table S2.** Data on expression stability of reference gene *F-box*.

| Experimental conditions | Mean Ct ± SD of three biological replicates |
| --- | --- |
| True leaf tissues |  |
| Wild type plants – Control | 22.307 ± 0.207 |
| AC transgenic plants – DEX treatment 0 h | 22.083 ± 0.322 |
| AC transgenic plants – DEX treatment 6 h | 21.707 ± 0.092 |
| AC transgenic plants – DEX treatment 24 h | 22.107 ± 0.228 |
| Aerial part tissues |  |
| Wild type plants – DEX treatment 0 h | 20.440 ± 0.027 |
| Wild type plants – DEX treatment 6 h | 20.547 ± 0.105 |
| AC transgenic plants – DEX treatment 0 h | 20.250 ± 0.238 |
| AC transgenic plants – DEX treatment 6 h | 20.247 ± 0.311 |


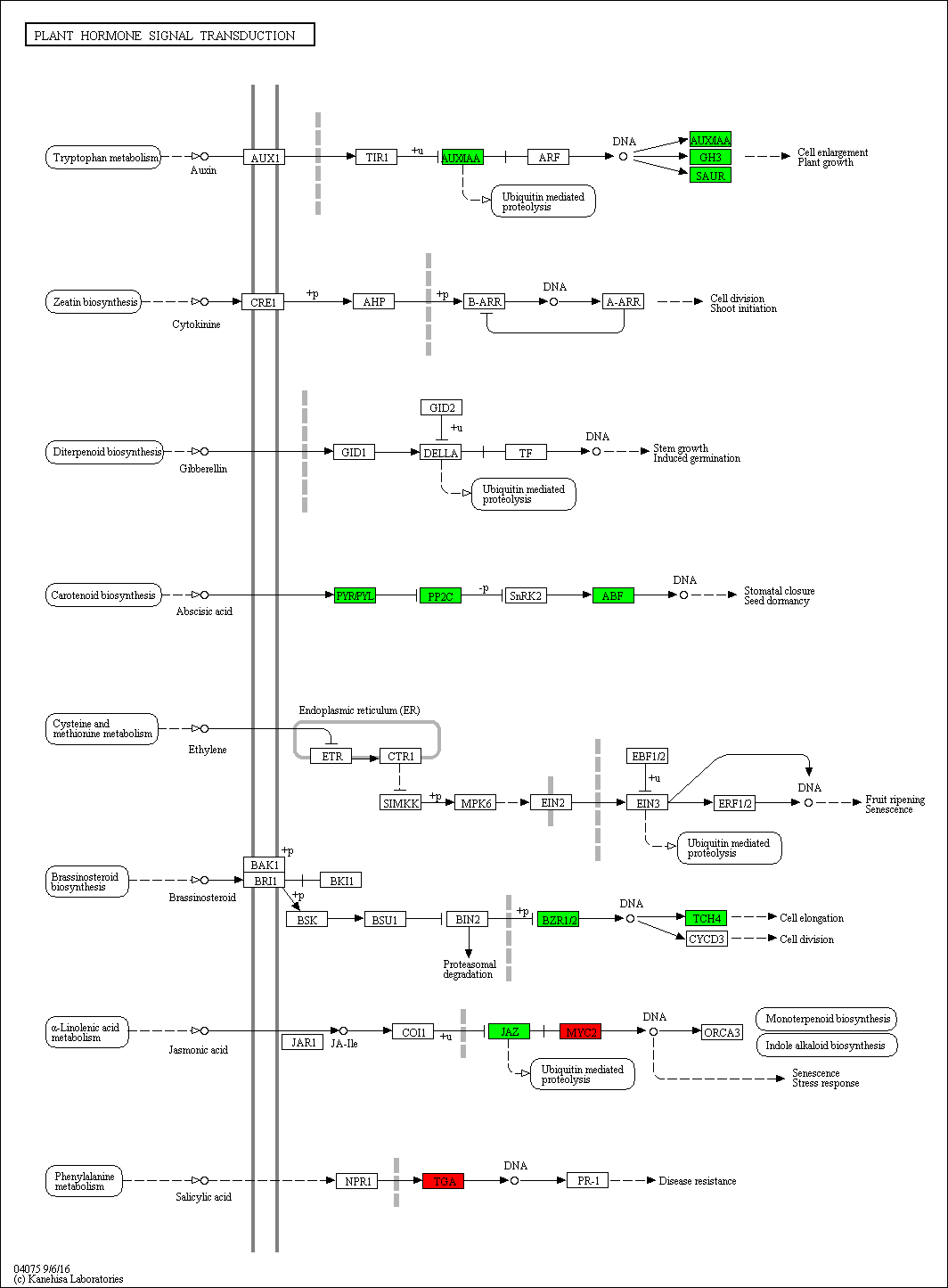


**Figure S1.** KEGG map of plant hormone signal transduction. Genes highlighted by red and green in the figure indicate the mapped DEGs of up- and down-regulation, respectively. KEGG, Kyoto Encyclopedia of Genes and Genomes (https://www.genome.jp/kegg/). DEGs, differential expression genes.
